# Irisin Improves Autophagy of Aged Hepatocytes via Increasing Telomerase Activity in Liver Injury

**DOI:** 10.1155/2020/6946037

**Published:** 2020-01-02

**Authors:** Jianbin Bi, Lifei Yang, Tao Wang, Jia Zhang, Teng Li, Yifan Ren, Mengzhou Wang, Xue Chen, Yi Lv, Rongqian Wu

**Affiliations:** ^1^National Local Joint Engineering Research Center for Precision Surgery & Regenerative Medicine, Shaanxi Provincial Center for Regenerative Medicine and Surgical Engineering, The First Affiliated Hospital of Xi'an Jiaotong University, Xi'an, Shaanxi Province, China; ^2^Department of Hepatobiliary Surgery, The First Affiliated Hospital of Xi'an Jiaotong University, Xi'an, Shaanxi Province, China

## Abstract

An aged liver has decreased reparative capacity during ischemia-reperfusion (IR) injury. A recent study showed that plasma irisin levels predict telomere length in healthy adults. The aim of the present study is to clarify the role of irisin, telomerase activity, and autophagy during hepatic IR in the elderly. To study this, hepatic IR was established in 22-month- and 3-month-old rats and primary hepatocytes were isolated. The results showed that the old rats exhibited more serious liver injury and lower levels of irisin expression, telomerase activity, autophagy ability, and mitochondrial function than young rats during hepatic IR. Irisin activated autophagy and improved mitochondrial function via increasing telomerase activity in aged hepatocytes. Inhibition of telomerase activity by BIBP1532 abolished the protective role of irisin in hepatocytes during hypoxia and reoxygenation. Additionally, this study proved irisin increased the telomerase activity via inhibition of the phosphorylation of JNK during hepatic IR. Administration of exogenous irisin significantly mitigated the inflammation, oxidative stress, apoptosis, and liver injury in an old rat model of hepatic IR. In conclusion, irisin improves autophagy of aged hepatocytes via increasing telomerase activity in hepatic IR. Irisin exhibits conspicuous benefits in increasing reparative capacity of an aged liver during hepatic IR.

## 1. Introduction

Liver transplantation is the only expectation for many patients with end-stage liver diseases, acute liver failure, and malignant tumors. Acceptance of aged livers is one of the important strategies to solve the shortage of donor organs [1]. Additionally, with the aging of the population, the number of elderly patients suffering from hepatic carcinoma and other liver diseases is increasing [2, 3]. Liver resection is the main treatment for these diseases. However, both elderly liver donors and elderly patients undergoing liver resection must accept higher surgical risks. Ischemia-reperfusion (I/R) is a major cause of detrimental liver injury following liver transplantation and liver resection [4]. Ischemia leads to energy supply crisis and hypoxic injury of hepatocytes, and worse still after reperfusion, excessive reactive oxygen species (ROS), massive inflammatory mediators, and vasoactive substances lead to mitochondrial injury, which ultimately leads to a large number of hepatocyte apoptosis and liver failure [5]. The aged liver has significantly decreased reparative capacity following IR compared with the young liver [6]. The mechanisms affecting the poor prognosis after liver IR of elderly patients include weaker hepatocyte autophagy and poorer mitochondrial function [7]. Therefore, improving autophagy and mitochondrial function can be a strategy to alleviate IR injury in elderly patients.

Autophagy is a self-protective response to cellular stress by removing damaged organelles or long-lived cytoplasmic proteins [8]. Impaired autophagy in the elderly liver leads to decreased tolerance of hepatocytes to IR injury [9]. Improving autophagy is an important therapeutic method to alleviate hepatic IR injury. For example, lithium prevented warm IR injury via increasing hepatocyte autophagy [10]. Decreased telomerase activity is one of the important signs of cell and organ aging and generates cellular growth arrest, senescence, and apoptosis [11]. Telomerase reverse transcriptase- (TERT-) deficient mice showed significant mitochondrial dysfunction and oxidative stress [11]. It has become a hot topic in tumor research that inhibition of the activity of telomerase promotes hepatocyte apoptosis [12]. Stress including genotoxic events causes phosphorylation of mitogen-activated protein kinase (MAPK), which in turn promotes cytoplasmic protein phosphorylation or translocates into the nucleus to inhibit transcription factor activity and TERT promoter function, further regulating of telomerase activity, survival, growth, and differentiation of cells [13–15]. For example, progesterone and EGF promoting telomerase activation depend on inhibition of the MAPK phosphorylation [16, 17]. Telomerase activity is positively correlated with autophagy ability [18]. However, whether autophagy is reduced in an elderly liver due to decreased telomerase activity after IR is still unknown.

Irisin is a newly defined exercise hormone associated with energy metabolism, glucose tolerance, and bone formation [19]. In addition, irisin is also related to mitochondrial function in IR injury [20]. Previous studies found that serum irisin levels in the elderly are significantly lower than those in the young [21] and plasma irisin levels are positively correlated with telomerase length, which indicates that irisin can predict telomere length in healthy adults [22]. Exercise can promote the secretion of irisin and reduce the incidence of atherosclerosis and cardiovascular diseases in the elderly [21, 23]. However, whether irisin plays a protective role by regulating telomerase activity has not been studied. Here, we hypothesized that irisin promotes autophagy by regulating telomerase activity, thereby protecting mitochondrial function and alleviating IR injury in the elderly liver. The main purpose of this study is to clarify the relationship among irisin, telomerase activity, and autophagy in liver IR injury and whether exogenous irisin could be used to prevent or treat liver IR injury in the elderly.

## 2. Materials and Methods

### 2.1. Experimental Animals

Male Sprague-Dawley rats were purchased from the Laboratory Animal Center of Xi'an Jiaotong University. All rats were housed (2 per cage) in clear, pathogen-free polycarbonate cages in the animal care facility (23°C, 12 h/12 h light/dark cycle, 50% humidity, and ad libitum access to food and water). Experiments were performed on male Sprague-Dawley rats (old group: weighing 500–650 g, aged 22 months; young group: weighing 250–300 g, aged 3 months). Animals were randomly allocated to each group. All animal experiments were performed in accordance with the guidelines of the China Council on Animal Care and Use and approved by the Institutional Animal Care and Use Committee of the Ethics Committee of Xi'an Jiaotong University Health Science Center, China (approval number: 2017-564). In this study, the rats were anesthetized by 3% isoflurane inhalation with an anesthesia machine and euthanasia was conducted by exsanguination under deep anesthesia. A total of 72 rats were used (*n* = 6 per group) in this experiment.

### 2.2. Hepatocyte Isolation, Culture, and Hypoxia/Reoxygenation (H/R)

Rat hepatocytes were isolated by Seglen's collagenase perfusion method [24]. Briefly, the rats were anaesthetized by inhaling 3% isoflurane. The liver was infused with preconditioning fluid for 10 min and type IV collagenase infusion solution containing 0.05% calcium for 6 min via portal vein intubation. Then, place the liver piece in a crystallizing dish containing 100-200 ml hepatocyte cleaning fluid, remove the Glisson's capsule carefully, and gently shake out the cells. The hepatocytes were filtered with a 100 *μ*m cell strainer (352360, Corning, USA). The viability after isolation, as judged by trypan blue exclusion, was 91.4 ± 0.53% and 86.2 ± 0.83% in young and old hepatocytes. Cells with the viability above 80% were used for further experiments. The isolated hepatocytes were cultured with RPMI 1640 medium containing 10% fetal bovine serum. The H/R model was performed in hepatocytes by exposing to hypoxia condition (94% N_2_, 5% CO_2_, and 1% O_2_) at 37°C in glucose/FBS free RPMI-1640 medium. One hour later, the hepatocytes returned to normal culture conditions. The sham group was treated with normal medium without hypoxia. The subsequent experiments were performed at 8 h after reoxygenation.

### 2.3. Rat Model of Hepatic I/R and Experimental Design

Partial (70%) liver warm I/R was performed as described previously [25]. In brief, rats were anaesthetized by inhaling 3% isoflurane. Seventy percent of liver arterial/portal venous blood was obstructed with a microvascular clip across the portal triad, above the right lateral lobe. Sham-operated rats underwent the same operation procedure without vascular occlusion. According to the requirements of the study, ischemia duration was 40 min and 60 min. The samples were collected at 24 h after reperfusion.

### 2.4. Administration of Irisin, Irisin-Neutralizing Antibody, and BIBR 1532

In an vivo study, irisin (067-29A, Phoenix Pharmaceuticals, USA) was administrated by intravenous injection in old rats (250 *μ*g/kg, single dose) at the beginning of reperfusion; irisin-neutralizing antibody (G-067-17, Phoenix Pharmaceuticals, USA) was administrated by intravenous injection in young rats (50 *μ*g/kg, single dose) at 24 h before hepatic IR; BIBR 1532 (S1186, Selleck, China) was administrated by intravenous injection in young rats (20 mg/kg, single dose) at the beginning of reperfusion. In an in vitro study, the concentrations of irisin, BIBR 1532, and anisomycin (S7409, Selleck, China) administration were 10 nm, 50 *μ*M, and 10 *μ*M, respectively, at the beginning of reoxygenation.

### 2.5. Histological Analysis

H&E staining of fixed liver tissues was performed as described previously [20]. The percentage of the liver necrosis area was quantified blindly in more than 5 fields for each rat. The liver histological score was calculated according to the following 6 items: cytoplasmic color fading, vacuolization, nuclear condensation, nuclear fragmentation, nuclear fading, and erythrocyte stasis. Each item is graded as follows: 0—no, 1—mild, 2—moderate, and 3—severe. The liver histological score was the sum of score for each item, ranging from 0 to 18 [20]. The transmission electron microscope (TEM) was used to observe the autophagosomes and mitochondria performed as described previously [20]. Representative fields were chosen for application.

### 2.6. TUNEL Fluorescence Staining

A TUNEL kit (11684795910, Roche, Switzerland) was used for TUNEL staining following the instruction. A representative field was chosen for application.

### 2.7. Flow Cytometry Analysis

An annexin V-FITC/PI apoptosis detection kit (AD10, Dojindo Laboratories, Shanghai, China) was used for apoptotic cell determination with a flow cytometry (ACEA Biosciences, Inc.) following the instruction. The apoptotic cell percentage was defined as the sum of the early and late apoptotic cell percentages.

### 2.8. qPCR

qPCR was performed as described previously [20]. The primers were synthesized by Takara Biomedical Technology (Beijing) as follows: Rattus norvegicus telomerase reverse transcriptase (TERT): forward 5′-GCTGGACACTCGGACTTTGGA-3′, reverse 5′-ACTTCAACCGCAAGACTGACAAGA-3′; Rattus norvegicus telomerase RNA component (TERC): forward 5′-ACCCTATTGTTATAGCTGTGGGTTC-3′, reverse 5′-CACCAGAGCTCCTACGCTGA-3′; Rattus norvegicus telomeric repeat binding factor 1 (TERF1): forward 5′-GACTACCCAGTCTTACAGCTTACCA-3′, reverse 5′-AGGGTGTAATCCGCTCATCAA-3′; Rattus norvegicus PPARG coactivator 1 alpha (PGC1*α*): forward 5′-TCAGAACAAACCCTGCCATTGTTA-3′, reverse 5′-AGGGTCATCGTTTGTGGTCAGATA-3′; Rattus norvegicus mitochondrial transcription factor A (TFAM): forward 5′-TGAAGCTTGTAAATCAGGCTTGGA-3′, reverse 5′-GAGATCACTTCGCCCAACTTCAG-3′; Rattus norvegicus nuclear respiratory factor 1 (NRF1): forward 5′-CACTCTGGCTGAAGCCACCTTAC-3′, reverse 5′-TCACGGCTTTGCTGATGGTC-3′. The results were normalized against Rattus norvegicus *β*-actin: forward 5′-GACTCATCGTACTCCTGCTTGCTG-3′; reverse, 5′-GGAGATTACTGCCCTGGCTCCTA-3′. The reaction conditions are as follows: first stage—95°C for 30 s; second stage—95°C for 5 s, 60°C for 30 s, and amplification for 40 cycles; and third stage—95°C for 15 s, 60°C for 1 min, and 95°C for 15 s. The relative levels were calculated using the comparative-Ct method (*ΔΔ*Ct method).

### 2.9. Western Blot Analysis

Western blot was performed as described previously [20]. The primary rabbit anti-irisin antibody (1 : 1000 dilution, ab174833, Abcam, USA); rabbit anti-TERT antibody (1 : 1000 dilution, ab32020, Abcam, USA); rabbit anti-LC3B antibody (1 : 1,000 dilution, 3868, Cell Signaling Technology, USA); rabbit anti-P62 antibody (1 : 1,000 dilution, 5114, Cell Signaling Technology, USA); rabbit anti-PGC1*α* antibody (ab54481, Abcam, USA, 1 : 200 dilution); rabbit anti-TFAM antibody (ab131607, Abcam, USA, 1 : 200 dilution); rabbit anti-P-p38 antibody (1 : 1,000 dilution, 4511s, Cell Signaling Technology, USA); rabbit anti-P-ERK antibody (1 : 1,000 dilution, 4370s, Cell Signaling Technology, USA); rabbit anti-P-JNK antibody (1 : 1,000 dilution, 4668s, Cell Signaling Technology, USA); MAPK family antibody sampler kit (1 : 1,000 dilution, 9926, Cell Signaling Technology, USA); and rabbit anti-*β*-actin antibody (1 : 1000 dilution, 4967, Cell Signaling Technology, USA) were incubated overnight at 4°C on a shaker. The secondary HRP-conjugated goat anti-rabbit IgG (1 : 2000 dilution, SA00001-2, Proteintech, China) was incubated for 1 h at room temperature. The protein quantification was performed by ImageJ2x software.

### 2.10. Enzyme-Linked Immunosorbent Assays (ELISA)

Serum irisin, tumor necrosis factor *α* (TNF-*α*), interleukin 6 (IL6), and interleukin 10 (IL10) were measured with an irisin ELISA kits (SEN576Ra, Cloud-Clone Corp. USCN Life Science, China), a TNF-*α* ELISA kit (CSB-E11987r, Cusabio, China), an IL6 ELISA kit (SEA079Ra, Cloud-Clone Corp. USCN Life Science, China), and an IL10 ELISA kit (SEA056Ra, Cloud-Clone Corp. USCN Life Science, China) following the instructions.

### 2.11. Determination of Serum ALT, Lactate, and LDH Levels

Serum alanine aminotransferase (ALT), lactate, and lactic dehydrogenase (LDH) levels were measured with an ALT assay kit (C009-2, Nanjing Jiancheng Bioengineering Institute, Nanjing, China), a lactate assay kit (A019-2, Nanjing Jiancheng Bioengineering Institute, Nanjing, China), and a LDH assay kit (A020-2, Nanjing Jiancheng Bioengineering Institute, Nanjing, China) following the instructions.

### 2.12. Determination of Oxidative Stress

The oxidation index malondialdehyde (MDA) and the reduction index superoxide dismutase (SOD) and glutathione peroxidase activity (GSH-Px) were detected to evaluate the oxidative stress state of the body. Liver homogenate was obtained and quantified using the BCA protein quantification kit (P0012S, Beyotime, Shanghai, China). MDA, SOD, and GSH-PX were measured with an MDA assay kit (A003-1, Nanjing Jiancheng Bioengineering Institute, Nanjing, China), SOD assay kit (A001-3, Nanjing Jiancheng Bioengineering Institute, Nanjing, China), and GSH-PX assay kit (A005, Nanjing Jiancheng Bioengineering Institute, Nanjing, China) following the instructions.

### 2.13. Statistical Analysis

All values were expressed as the means ± standard error of the mean (SEM). A *t*-test was used to analyze the differences between two groups, and one-way ANOVA (Student-Newman-Keuls) was applied to analyze the differences among three or more groups by SPSS 18.0. *P* < 0.05 represents a significant difference.

## 3. Results

### 3.1. The Old Rats Showed More Serious Liver Injury Than Young Rats during Hepatic IR

**Figure 1:**
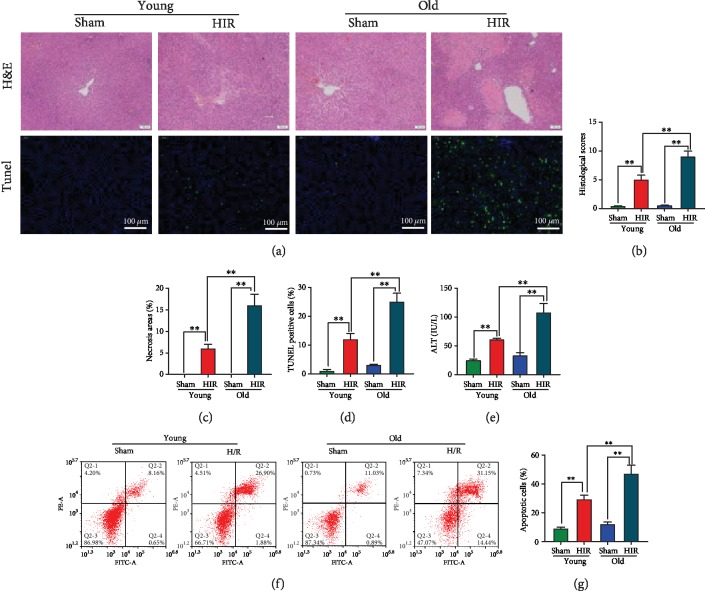
The old rats showed more serious liver injury than young rats during hepatic IR. Partial (70%) liver arterial/portal venous blood was interrupted for 40 minutes in 3-month- and 22-month-old rats. Blood and liver samples were harvested at 24 h after reperfusion. (a) H&E and TUNEL (green) staining of the liver. (b) Liver histological scores. (c) Percentage of necrotic areas. (d) Percentage of TUNEL-positive cells. (e) Serum ALT. Rat hepatocytes were isolated, and the hypoxia/reoxygenation (H/R) model was performed in hepatocytes by exposing to hypoxia condition (94% N_2_, 5% CO_2_, and 1% O_2_) at 37°C in glucose/FBS-free RPMI-1640 medium. One hour later, the hepatocytes returned to normal culture conditions. The sham group was treated with normal medium without hypoxia. The subsequent experiments were performed at 8 h after reoxygenation. (f, g) Flow cytometry analysis of hepatocyte apoptotic percentage. *n* = 6; mean ± SEM; ^∗^*P* < 0.05 and ^∗∗^*P* < 0.01.

H&E staining of the liver showed marked necrosis and inflammatory cell infiltration at 24 h after hepatic IR. Meanwhile, larger areas of necrosis and higher liver injury score were gained in old rats compared with the young rats (Figures 1(a)–1(c)). TUNEL staining of apoptotic cells revealed the old rats had higher percentages of hepatocyte apoptosis than the young rats after hepatic IR (11.8 ± 2.1% vs. 24.8 ± 3.1%, Figures 1(a) and 1(d)). Furthermore, the ALT levels of the young and old rats increased by 2.45-fold and 3.3-fold after hepatic IR, respectively, and the elderly rats exhibited higher serum ALT levels in contrast to the young rats (Figure 1(e)). Primary hepatocytes were isolated from young and old rats. Hepatocytes were treated with hypoxia (94% N_2_, 5% CO_2_, 1% O_2_, and glucose/FBS free) and reoxygenation. The sham group was treated with normal medium without hypoxia. Consistent with the in vivo study, flow cytometry analysis showed that the apoptotic percentage of aged hepatocytes was as high as 46.7 ± 5.8%, which is much higher than that of young hepatocytes after H/R treatment (Figures 1(f) and 1(g)).

### 3.2. The Old Rats Showed More Serious Decrease of Irisin Expression, Telomerase Activity, Autophagy Ability, and Mitochondrial Function Than Young Rats during Hepatic IR

**Figure 2:**
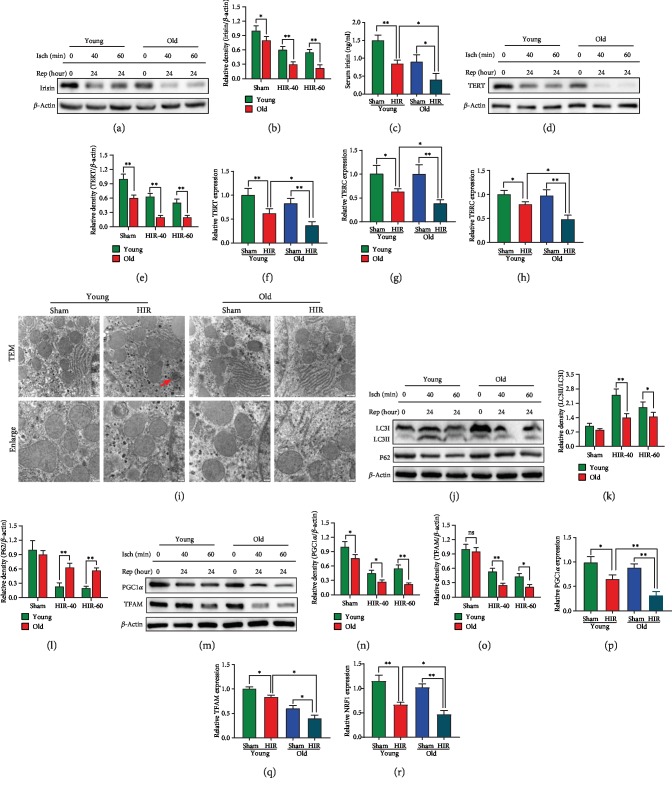
The old rats showed more a serious decrease of irisin expression, telomerase activity, autophagy ability, and mitochondrial function than young rats during hepatic IR. Partial (70%) liver arterial/portal venous blood was interrupted for 40 and 60 minutes in 3-month- and 22-month-old rats. Liver samples were harvested at 24 h after reperfusion. (a, b) Western blot analysis of liver irisin expression. (c) Serum irisin levels. (d, e) Western blot analysis of liver telomerase reverse transcriptase (TERT) expression. (f–h) qPCR analysis of liver *TERT*, *TERC*, and *TERF1* expression. (i) TEM analysis. The red arrow indicates autophagosomes. (j–l) Western blot analysis of liver LC3B and P62 expression. (m–o) Western blot analysis of liver PGC1*α* and TFAM expression. (p–r) qPCR analysis of liver *PGC1α*, *TFAM*, and *NRF1* expression. *n* = 6; mean ± SEM; ^∗^*P* < 0.05 and ^∗∗^*P* < 0.01.

To investigate the mechanisms that the elderly was more sensitive to hepatic ischemia reperfusion, irisin expression, telomerase activity, autophagy ability, and mitochondrial function were detected. The results showed that liver irisin levels were decreased in sham-treated, 40 min ischemia-treated, and 60 min ischemia-treated old rats compared with the corresponding group of young rats at 24 h after reperfusion (Figures 2(a) and 2(b)). Meanwhile, serum irisin levels were decreased by 43.3% and 61.7% in the young and old rats after hepatic IR, respectively. The concentration of irisin in the young rats was 2.1 times higher than that in the older rats at 24 h after reperfusion (Figure 2(c)). Besides, Western blot showed that the old rats have lower TERT levels compared with the young rats in both the sham-operated and IR-operated groups (Figures 2(d) and 2(e)). qPCR analysis of *TERT*, *TERC*, and *TERF1* also revealed the liver telomerase activity was decreased after hepatic IR and the elderly showed more severe declines (Figures 2(f)–2(h)). Furthermore, TEM analysis showed that autophagosomes increased significantly in young rats, but not in old rats during hepatic IR (Figure 2(i)). Consistent with the TEM analysis, Western blot analysis of LC3B and P62 showed the autophagy of the older rats was worse than that of the younger rats after hepatic IR (Figures 2(j)–2(l)). Moreover, a prominent reduction of mitochondrial function-related PGC1*α* and TFAM was observed in the old rats compared with the young rats both in the sham-operated and IR-operated groups (Figures 2(m)–2(o)). qPCR analysis showed the similar results that the elderly had worse mitochondrial function (Figures 2(p)–2(r)).

### 3.3. Irisin Improved Telomerase Activity, Autophagy, and Mitochondrial Function in Old Rats after Hepatic IR

**Figure 3:**
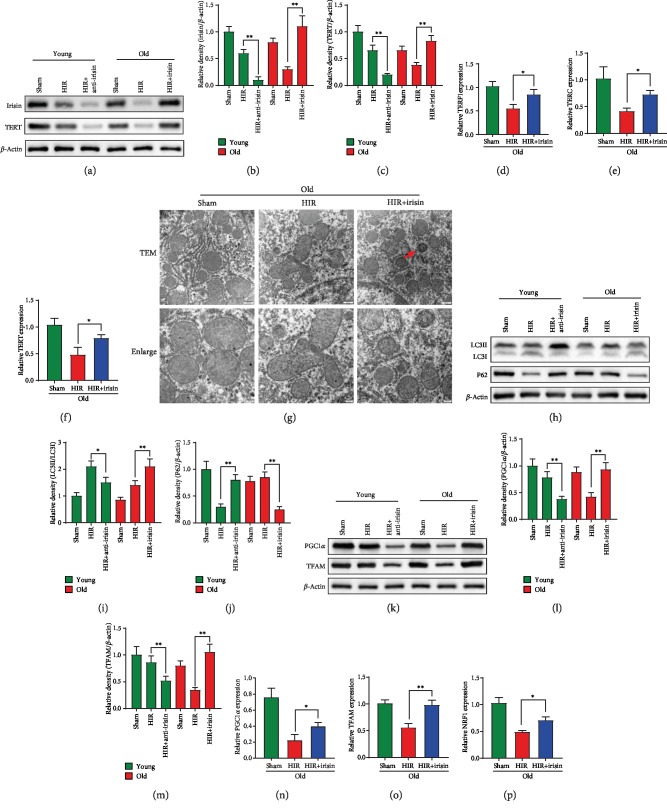
Irisin improved telomerase activity, autophagy, and mitochondrial function in old rats after hepatic IR. Partial (70%) liver arterial/portal venous blood was interrupted for 40 minutes in 3-month- and 22-month-old rats. Irisin was administrated in old rats (iv. 250 *μ*g/kg, single dose) at the beginning of reperfusion. Irisin-neutralizing antibody was administrated in young rats (iv. 50 *μ*g/kg, single dose) at 24 h before hepatic IR. Liver samples were harvested at 24 h after reperfusion. (a–c) Western blot analysis of liver irisin and TERT expression. (d–f) qPCR analysis of liver *TERF1*, *TERC*, and *TERT* expression in old rats. (g) TEM analysis. The red arrow indicates autophagosomes. (h–j) Western blot analysis of liver LC3B and P62 expression. (k–m) Western blot analysis of liver PGC1*α* and TFAM expression. (n–p) qPCR analysis of liver *PGC1α*, *TFAM*, and *NRF1* expression; *n* = 6; mean ± SEM; ^∗^*P* < 0.05 and ^∗∗^*P* < 0.01.

To clarify the role of irisin in telomerase activity, autophagy, and mitochondrial function, the young rats were pretreated with irisin-neutralizing antibody and old rats received exogenous irisin treatment. We found that the irisin-neutralizing antibody pretreating young rats showed lower irisin and TERT expression after hepatic IR. Meanwhile, old rats receiving exogenous irisin had significantly higher TERT expression after hepatic IR (Figures 3(a)–3(c)). qPCR analysis showed that *TERT*, *TERC*, and *TERF1* increased by 1.63-fold, 1.78-fold, and 1.56-fold after irisin treatment during hepatic IR (Figures 3(d)–3(f)). Furthermore, exogenous irisin-treated old rats exhibited more autophagosomes than vehicle-treated rats during hepatic IR (Figure 3(g)). Western blot analysis of LC3B and P62 also revealed irisin increased autophagy ability of old rats during hepatic IR. Meanwhile, young rats pretreated with irisin-neutralizing antibody significantly demonstrated decreased autophagy ability after hepatic IR (Figures 3(h)–3(j)). Similar to the results of autophagy, irisin-neutralizing antibody inhibited the mitochondrial function after hepatic IR in young rats. More importantly, the exogenous irisin markedly improved mitochondrial function after hepatic IR in old rats (Figures 3(k)–3(p)).

### 3.4. Inhibition of Telomerase Activity by BIBP1532 Abolished the Protective Role of Irisin in Increasing Autophagy and Mitochondrial Function during Hepatic H/R

**Figure 4:**
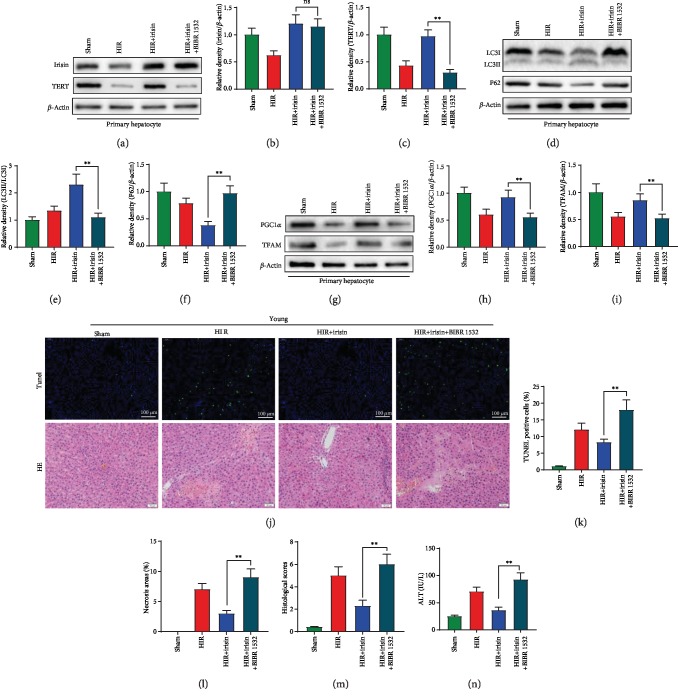
Inhibition of telomerase activity by BIBP1532 abolished the protective role of irisin in increasing autophagy and mitochondrial function during hepatic H/R. Old rat hepatocytes were isolated, and the hypoxia reoxygenation (H/R) model was performed in hepatocytes by exposing to hypoxia condition (94% N_2_, 5% CO_2_, and 1% O_2_) at 37°C in glucose/FBS-free RPMI-1640 medium. One hour later, the hepatocytes returned to normal culture conditions. The sham group was treated with normal medium without hypoxia. BIBR 1532 was administrated at the beginning of reoxygenation (50 *μ*M). The subsequent experiments were performed at 8 h after reoxygenation. (a–c) Western blot analysis of liver irisin and TERT expression. (d–f) Western blot analysis of liver LC3B and P62 expression. (g–i) Western blot analysis of liver PGC1*α* and TFAM expression. Partial (70%) liver arterial/portal venous blood was interrupted for 40 minutes in 3-month- and 22-month-old rats. Blood samples were harvested at 24 h after reperfusion. Partial (70%) liver arterial/portal venous blood was interrupted for 40 minutes in 3-month-old rats. Irisin (iv. 250 *μ*g/kg, single dose) and BIBR 1532 (iv. 20 mg/kg, single dose) were administrated in young rats at the beginning of reperfusion. (j) TUNEL (green) and H&E staining of the liver. (k) Percentage of TUNEL-positive cells. (l) Percentage of necrotic areas. (m) Liver histological scores. (n) Serum ALT. *n* = 6; mean ± SEM; ^∗^*P* < 0.05 and ^∗∗^*P* < 0.01.

To determine the role of telomerase in irisin-induced improvement of autophagy and mitochondrial function, BIBP1532, a specific telomerase inhibitor, was administrated in aged primary hepatocytes and young rats. In aged primary hepatocytes, BIBP1532 treatment had no effect on irisin expression but significantly decreased the TERT expression after hepatic H/R (Figures 4(a)–4(c)). Additionally, BIBP1532 abolished the protective role of irisin in increasing autophagy and mitochondrial function in aged hepatocytes (Figures 4(d)–4(i)). In an in vivo study, BIBP1532 significantly aggravated the liver injury, increased hepatocyte apoptosis, area of liver necrosis, histological score, and serum ALT levels by 119.5%, 196.4%, 166.1%, and 156.3% in contrast to the irisin-treated group (Figures 4(j)–4(n)).

### 3.5. Irisin Activated the Telomerase Activity via Inhibition of the Phosphorylation of JNK during Hepatic H/R

**Figure 5:**
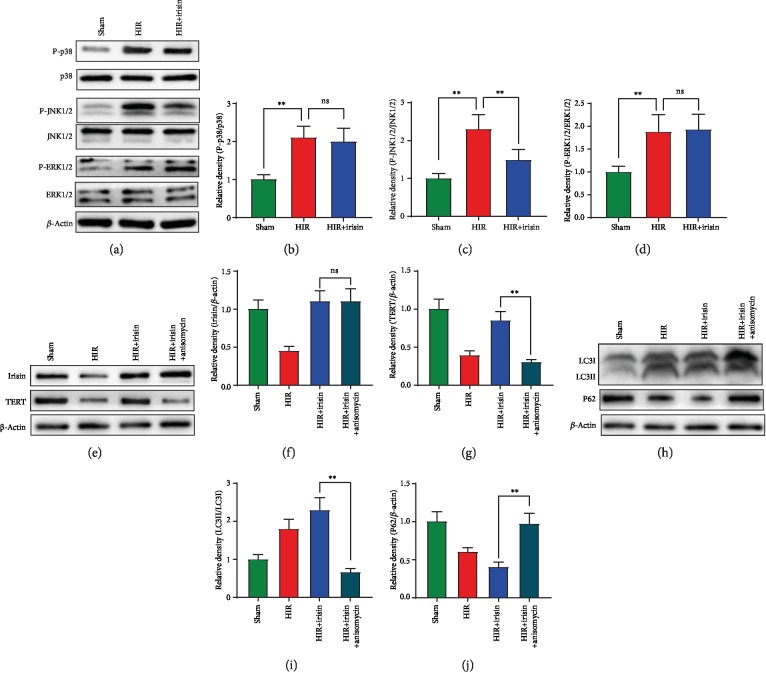
Irisin activated the telomerase activity via inhibition of the phosphorylation of JNK during hepatic H/R. Old rat hepatocytes were isolated, and the hypoxia reoxygenation (H/R) model was performed in hepatocytes by exposing to hypoxia condition (94% N_2_, 5% CO_2_, and 1% O_2_) at 37°C in glucose/FBS-free RPMI-1640 medium. One hour later, the hepatocytes returned to normal culture conditions. The sham group was treated with normal medium without hypoxia. Anisomycin was administrated at the beginning of reoxygenation (10 *μ*M). The subsequent experiments were performed at 8 h after reoxygenation. (a–d) Western blot analysis of liver P-p38/p38, P-JNK/JNK, and P-ERK/ERK expression. (e–g) Western blot analysis of liver irisin and TERT expression. (h–j) Western blot analysis of liver LC3B and P62 expression. *n* = 6; mean ± SEM; ^∗^*P* < 0.05 and ^∗∗^*P* < 0.01.

The MAPK pathways are closely related to telomerase activity. The MAPK family was detected to clarify how irisin upregulated telomerase activity. As shown in Figures 5(a)–5(d), phosphorylation of p38, JNK, and ERK was significantly increased after hepatic H/R model. Irisin treatment showed no difference in phosphorylation of p38 and ERK but remarkably decreased the P-JNK levels. To further confirm the role of P-JNK in irisin-induced increase of telomerase activity, anisomycin, a JNK MAPK inhibitor, was administrated in aged primary hepatocyte. Anisomycin treatment had no effect on irisin expression but significantly decreased the TERT expression after hepatic H/R (Figures 5(e)–5(g)). Additionally, anisomycin abolished the protective role of irisin in increasing autophagy (Figures 5(h)–5(j)).

### 3.6. Irisin Mitigates Liver Injury in Old Rats after Hepatic IR

**Figure 6:**
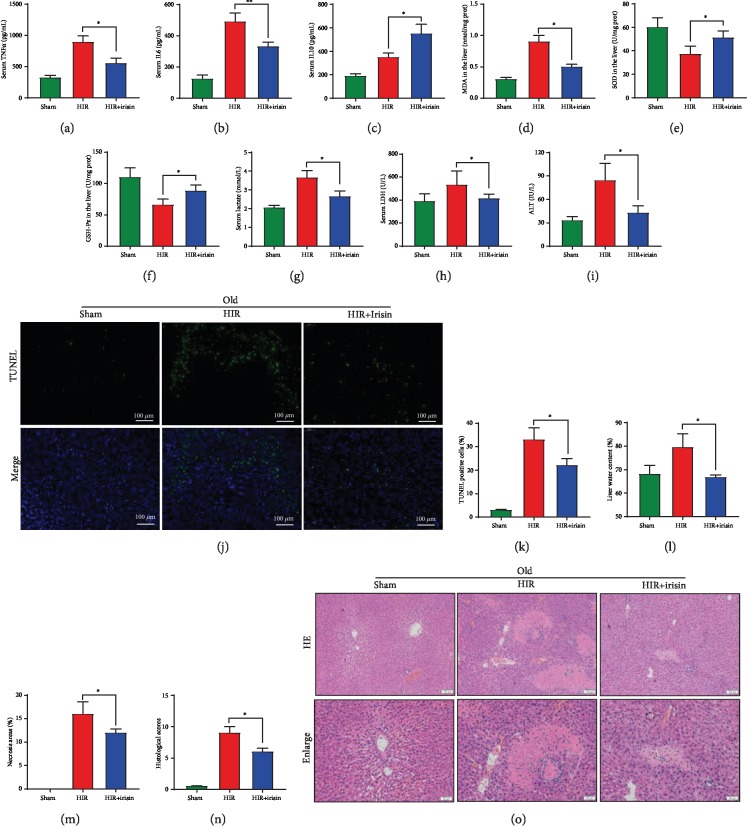
Irisin mitigates liver injury in old rats after hepatic IR. Partial (70%) liver arterial/portal venous blood was interrupted for 40 minutes in 22-month-old rats. Irisin was administrated in old rats (iv. 250 *μ*g/kg, single dose) at the beginning of reperfusion; Samples were harvested at 24 h after reperfusion. (a–c) Serum TNF*α*, IL6, and IL10 levels. (d–f) Serum MDA, SOD, and GSH-PX levels. (g–i) Serum lactate, LDH, and ALT levels. (j, k) TUNEL (green) staining of the liver and percentage of TUNEL-positive cells. (l) Liver water content. (m) Percentage of necrotic areas. (n) Liver histological scores. (o) H&E staining of the liver. *n* = 6; mean ± SEM; ^∗^*P* < 0.05 and ^∗∗^*P* < 0.01.

ELISA results showed that irisin significantly decreased the serum levels of TNF*α* and IL6 by 38.2% and 32.7%, respectively, and increased the anti-inflammatory cytokine IL10 by 57.1% at 24 h after hepatic IR in old rats (Figures 6(a)–6(c)). Furthermore, we found that irisin reduced the liver MDA level and increased the antioxidant indices SOD and GSH-Px after hepatic IR (Figures 6(d)–6(f)). Additionally, irisin improved the liver metabolism that serum lactate was reduced after irisin administration (Figure 6(g)). More importantly, the liver functions were improved after irisin treatment that serum LDH and ALT were significantly decreased by 22.3% and 49.4% at 24 h after hepatic IR in old rats, respectively (Figures 6(h) and 6(i)). TUNEL staining of apoptotic cells showed irisin markedly decreased the percentage of apoptotic cells (Figures 6(j) and 6(k)). Moreover, exogenous irisin-treated old rats exhibited less water content, milder liver injury, smaller necrosis area, and lower histological scores compared with the vehicle-treated old rats after at 24 h after hepatic IR (Figures 6(l)–6(o)).

## 4. Discussion

In this study, we initially discovered that irisin activated autophagy via increasing telomerase activity in aged hepatocytes after hepatic IR. The further study proved irisin increased the telomerase activity via inhibition of the phosphorylation of JNK during hepatic IR. Exogenous irisin significantly mitigated the inflammation, oxidative stress, apoptosis, and liver injury in old rat model of hepatic IR. Irisin exhibited conspicuous benefits in increasing reparative capacity of an aged liver during hepatic IR.

IR injury is an important mechanism of tissue and organ injury that occurs after circulatory disorders such as traumatic shock, surgery, and organ transplantation [26]. The injury mechanism of IR is approximately the same in organs such as the heart, kidney, and brain [26]. Tissue ischemia leads to energy supply crisis and hypoxic injury, which is a lethal cause of many diseases, such as myocardial infarction and stroke due to coronary atherosclerosis [27]. In a reperfusion phase, excessive reactive oxygen species (ROS), calcium overload, massive inflammatory mediators, and vasoactive substances lead to mitochondrial injury, which ultimately leads to tissue damage and organ dysfunction [28]. Oxygen free radical scavengers, Ca^2+^ blockers, and ischemic preconditioning have been shown to be effective in preventing and treating ischemia-reperfusion injury [26, 29, 30]. Irisin has antioxidant and mitochondrial protective effects [20]. This study is aimed at illuminating the effects of irisin on telomerase activity and autophagy during IR in the aged liver.

Acceptance of aged livers is one of the important strategies to solve the shortage of donor organs. About 30 percent of liver transplantations are conducted with using grafts aging more than 50 years over the past five years in the United States [1]. However, recipients transplanted with aged grafts showed more complications and shorter long-term survival [1]. Although the quality of elderly graft can be strictly evaluated to exclude cirrhosis, fatty liver, and cold ischemia time longer than 8 h, there is still no strategies to reduce the fact that old grafts are more sensitive to ischemia-reperfusion [1, 31, 32]. Additionally, with the aging of the population, the number of elderly patients suffering from hepatic carcinoma and other liver diseases is increasing [2, 3]. Liver resection is the main treatment for these diseases. Both elderly liver donors and elderly patients undergoing liver resection must accept higher surgical risks. Consistent with previous clinical studies [32, 33], we found that the old rats exhibited larger areas of necrosis, higher liver injury score, and worse liver function than young rats during hepatic IR. Thus, it is of great urgency to explore the specific mechanisms and treatment options of hepatic IR in the aged liver. Irisin, a newly defined hormone, is associated with many physiological processes such as energy metabolism, glucose tolerance, and bone formation [19, 34, 35]. The serum irisin level is modulated by exercise, diet, obesity, and some pathological conditions [36, 37]. Numerous studies have found that irisin plays protective roles in many diseases, such as diabetes, cardiovascular disease, and metabolic diseases [38–40]. Some studies found that the serum irisin level in the elderly was significantly lower than that in the young [21] and plasma irisin levels are positively correlated with telomerase length, which indicates that irisin can predict telomere length in healthy adults [22]. Additionally, irisin reduces the incidence of age-related atherosclerosis and cardiovascular diseases in the elderly [23]. Our previous study found that irisin is also related to mitochondrial function in ischemia-reperfusion injury [20]. However, whether irisin plays protective roles in regulating telomerase activity, autophagy, and IR injury of aged hepatocytes has not been studied. In this study, we initially discovered that irisin activated autophagy via increasing telomerase activity in aged hepatocytes after hepatic IR. Irisin exhibited conspicuous benefits in increasing reparative capacity of an aged liver during hepatic IR.

Autophagy is a self-protective response to cellular stress by removing damaged organelles or long-lived cytoplasmic proteins. There are two controversial opinions regarding autophagy in the hepatic IR. Some scholars believe that autophagy is one of the causes of death of hepatocyte, while others believe that autophagy is the process of self-protection [7]. Autophagy degrades damaged mitochondria and other nonfunctional organelles to maintain the energy supply and reduce oxygen free radical production. Some studies have shown that older livers are less capable of autophagy [9]. Wang et al. found that loss of autophagy-related protein, Atg4B, significantly increases the sensitivity of an aged liver to I/R injury [9]. Enhancing the autophagy ability of the aged liver may be the key to alleviate the ischemia reperfusion in the elderly. For example, lithium prevented warm IRI via increased hepatocyte autophagy [10]. Our study showed the old rats had weaker autophagy ability than young rats during hepatic IR and increased autophagy by exogenous irisin administration significantly alleviated the liver IR injury.

One of the remarkable findings of the present study is that irisin improves autophagy of aged hepatocytes via increasing telomerase activity in hepatic IR. Telomerase is a reverse transcriptase that synthesizes telomere sequences and is responsible for telomere elongation. Telomerase activity plays a decisive role in cell growth and apoptosis. A large number of studies have shown decreased telomerase activity in the elderly, which may be related to the development of many age-related diseases [41–43]. A previous study has showed increased telomerase significantly improving the repair and regeneration of cardiovascular tissue [44]. Besides, in tumor research field, inhibition of telomerase activity is a promising strategy to inhibit tumor development and promote tumor cell apoptosis [45]. A previous study showed irisin expression is upregulated in cancerous livers in contrast to the healthy livers and irisin significantly increased proliferation, invasion, and migration of liver cancer cells [46]. This phenomenon may be associated with the fact that irisin activates telomerase activity and thus promotes the tumor development. A further study of the effects of irisin on tumor should be studied in the future. Furthermore, the research reported that telomerase plays a protective role in neonatal hypoxic ischemic brain injury [47]. Increasing telomerase activity may be an important strategy to improve ischemia-reperfusion injury. Some studies have shown that telomerase can regulate autophagy, thereby affecting cells' response to oxidative stress [18]. Additionally, plenty of evidence suggested that the MAPK pathway regulates the activity of telomerase [16, 17, 48]. In this study, we found telomerase activity was significantly decreased after hepatic IR. A further study revealed irisin activated the telomerase activity via inhibition of the phosphorylation of JNK during hepatic IR. Irisin exhibited conspicuous benefits in increasing reparative capacity of an aged liver by increasing telomerase activity during hepatic IR.

Some limitations need to be noted in this study. First of all, ischemia-reperfusion injury in the elderly is a complex process. Our present study mainly focused on the effects of irisin on telomerase activity and autophagy in the aged liver after hepatic IR, and other mechanisms of irisin in hepatic IR need further exploration. Then, this study used only three indicators (MDA, SOD, and GSH-Px) to assess oxidative stress in vitro. In future studies, we will use a holistic approach for the evaluation of oxidative stress levels both in vitro and in vivo. Furthermore, this study showed irisin achieved great therapeutic effects on hepatic I/R injury in the aged liver; our results were only based on basic experiments and prospective clinical studies are needed.

## 5. Conclusions

Irisin improves autophagy of aged hepatocytes via increasing telomerase activity in hepatic IR. Irisin exhibits conspicuous benefits in increasing reparative capacity of an aged liver during hepatic IR.

## Data Availability

Data used to support the findings of this study are available from the corresponding author upon request.
